# Gefitinib-induced interstitial pneumonia: A case report and review of the literature

**DOI:** 10.3892/etm.2014.1495

**Published:** 2014-01-21

**Authors:** CHANGQIN LUO, MEILING LV, YUYAO LI, PEIJUN LIU, JIN YANG

**Affiliations:** 1 Department of Clinical Oncology, The First Affiliated Hospital of Xi’an Jiaotong University College of Medicine, Xi’an, Shaanxi 710061, P.R. China; 2 Center for Translational Medicine, The First Affiliated Hospital of Xi’an Jiaotong University College of Medicine, Xi’an, Shaanxi 710061, P.R. China

**Keywords:** gefitinib, interstitial pneumonia, glucocorticoid

## Abstract

The aim of this study was to explore the clinical characteristics of and treatment strategies for interstitial pneumonia induced by gefitinib in patients with advanced non-small cell lung cancer (NSCLC). The detailed clinical data of one patient with NSCLC and gefitinib-induced interstitial pneumonia were compiled and a review of relevant previous studies was performed. Based on this case report and the review, the clinical characteristics, mechanisms and treatment strategies of this rare disease were analyzed. The analyses showed that older, male patients with a long smoking history, high smoking index and adenocarcinoma (particularly bronchoalveolar carcinoma) were more likely to suffer from interstitial pneumonia while taking gefitinib. The onset time of interstitial pneumonia was 1–2 months subsequent to gefitinib administration. The clinical manifestations included chest tightness, shortness of breath, progressive dyspnea, severe hypoxemia and respiratory failure. Diffuse infiltration and alveolar interstitial shadows were observed on the chest tomography scan. In such circumstances, a timely judgment is required, in addition to the withdrawal of gefitinib treatment and the administration of high-dose glucocorticoids, as well as oxygen inhalation and anti-infective therapies, in order to relieve the symptoms. In conclusion, following the onset of gefitinib-induced interstitial pneumonia, the discontinuation of gefitinib is likely to alleviate the suffering of the majority of patients. Early interstitial pneumonia is not an absolute index for the permanent discontinuation of gefitinib treatment. It is necessary to comprehensively consider the benefits and hazards of gefitinib for the patients.

## Introduction

Gefitinib, an epidermal growth factor receptor (EGFR) type 1 tyrosine kinase inhibitor, blocks the signal transduction pathway implicated in the proliferation and survival of cancer cells (1). The use of gefitinib to treat patients with advanced non-small-cell-lung cancer (NSCLC) has raised significant concern among physicians. Gefitinib is better-tolerated and less toxic than conventional cytotoxic drugs; however, gefitinib-induced interstitial lung disease (ILD) has been demonstrated to be a serious adverse effect (2). Phase II studies showed that the objective response rates achieved after gefitinib treatment were between 10 and 20%, with minimal toxicities; mainly an acne-like rash and mild diarrhea, in patients with recurrent non-small cell lung cancer (the IDEAL trials) (3). In subsequent phase III trials, the addition of gefitinib to standard platinum-based chemotherapy failed to demonstrate a survival advantage in patients with untreated non-small cell lung cancer (the INTACT trials) (4,5). The worldwide incidence of ILD was approximately 1% (2% in the Japanese post-marketing experience and approximately 0.3% in a United States expanded access program). The median time to onset of ILD was 24 days in the Japan group and 42 days in the United States group. Approximately one-third of all ILD cases caused by gefitinib have been fatal (6). In this study, a case of gefitinib-induced interstitial pneumonia is described and previous case reports between 2003 and 2011 are reviewed, with the aims of summarizing the clinical features, mechanisms and treatment strategies of gefitinib-induced interstitial pneumonia and providing a reference for medication safety in the clinical treatment of NSCLC.

## Case report

**Figure 1:**
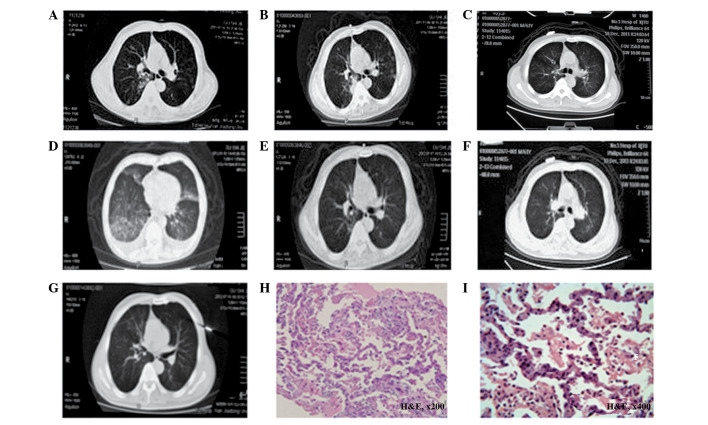
Primary lung tumor and interstitial pneumonia prior to and following treatment. (A) Prior to chemotherapy, multiple nodules were scattered in the double-lung field. (B) Following four cycles of chemotherapy, the therapeutic effect was progressive disease; (C) 30 days subsequent to the last administration of gefitinib, the therapeutic effect was partial response; (D) 60 days subsequent to the last administration of gefitinib, interstitial pneumonia occurred. (E) Following seven days of treatment, interstitial pneumonia was significantly relieved. (F) Results following 30 days of treatment; (G) results following one year of gefitinib treatment. (H and I) Lung pathology following interstitial pneumonia, occurring 60 days subsequent to the last administration of gefitinib [hematoxylin and eosin staining; magnification, ×200 (H) and ×400 (I)].

A 62-year-old man, complaining of fever and shortness of breath >1 year subsequent to surgery for right-sided NSCLC, was admitted to the First Affiliated Hospital of Xi’an Jiaotong University (Xi’an, China). In July 2010, the patient was revealed to have a lesion in the right upper lobe of the lung in a physical examination. In August 2010, upper right lobe resection and mediastinal lymph node dissections were performed. The tumor measured 3.5×3.0×2.0 cm. The pathological analysis of the resected upper right lung showed that the patient was suffering from differentiated adenocarcinoma and bronchioloalveolar carcinoma. The lung membrane, bronchial stump and lung hilar lymph nodes were not invaded (stage IB, pT2aN0M0). Following surgery, the patient did not receive any further treatment. In June 2011, the patient started to cough with obvious incentive; white phlegm was apparent, accompanied by intermittent bloody sputum. A chest computed tomography (CT) scan (Fig. 1A) showed multiple small nodules scattered throughout the patient’s lungs, suggesting pulmonary metastasis. The patient developed a progressive disease following four cycles of chemotherapy with a pemetrexed-cisplatin regimen, starting in July 2011 (Fig. 1B). The EGFR gene mutation test showed a deletion in exon 19; however, there were no mutations in exons 18, 20 or 21. The K-RAS gene mutation test showed no mutations in exons 13 or 61. In November 2011, daily treatment with oral gefitinib (250 mg/day) was initiated and the patient achieved a partial response one month subsequently (Fig. 1C). However, 60 days subsequent to the initiation of the gefitinib treatment, the patient developed fever, an aggravated dry cough and dyspnea. On the visit to our clinic, the patient appeared acutely ill and presented with dyspnea, with a body temperature fluctuating between 37.6 and 38.8°C. The patient was previously healthy and reported no history of chronic diseases, such as hypertension, heart disease and diabetes, and acute or chronic infection, such as hepatitis and tuberculosis. He had a smoking history of >20 years (40 cigarettes a day) and had quit smoking for 13 years, with a smoking index of 800. Physical examination showed that the patient had a body temperature of 38.3°C, a pulse rate of 124 beats/min, a respiratory rate of 41 breaths/min and a blood pressure of 130/80 mmHg. Hypoxemia and an oxyhemoglobin saturation of 88% were detected using pulse oximetry and visible, large erythema and pimples, with scales and itching, were observed around the ankles and the dorsal surfaces of the elbow joint. The examination results are listed in Table I. A chest CT scan showed interstitial inflammation of the lungs, bilateral pleural effusion and an adverse swelling of the lungs (Fig. 1D). The patient was admitted with suspected gefitinib-induced interstitial pneumonitis.

Following admission, gefitinib administration was discontinued immediately. No evidence of infection or the presence of pathological microorganisms was found in either the sputum or blood culture (Table I), while chest CT revealed a diffuse ground-glass opacity over the whole right and left lungs. A lung biopsy revealed interstitial pneumonia, with a widening of the alveolar septa and alveolar epithelial hyperplasia. A small quantity of inflammatory exudate was observed in the alveolar cavity (Fig. 1H and I). The patient was treated with inhaled oxygen, electrocardiography, cefoperazone/sulbactam (3.0 g twice daily; to provide resistance to infection), doxofylline (0.2 g once daily; to relieve coughing), high-dose methylprednisolone (1,000 mg daily for three days), inhalation of expectorant and dexamethasone (5.0 mg twice daily), acetylcysteine (600 mg/Tid; to prevent pulmonary fibrosis) and an infusion of protein. Therapies for the protection of the liver and gastric mucosa, improvement of the immune system, prevention of fungal infections (fluorouracil mouthwash) and adjustment of the intestinal flora were also administered to the patient. The patient’s dyspnea and hypoxemia improved significantly in three days. The results of the laboratory tests taken 10 days subsequent to the treatments are presented in Table I. The follow-up high-resolution CT scan (Fig. 1E) showed the total resolution of the ground-glass lesions and the absorption of the right-side pleural fluid. Based on the patient’s recovery, gefitinib treatment was reinitiated, under close observation. The gefitinib dosage was adjusted to 250 mg, taken once every two days. In addition, acetylcysteine was administered to prevent pulmonary fibrosis. No significant adverse response was detected in the patient. One month later, the follow-up chest CT (Fig. 1F) showed no interstitial pneumonia recurrence or tumor progression.

### Literature review

Based on the case reports and literature reviews on interstitial pneumonia published in Western countries and China between 2003 and 2011, the clinical characteristics of gefitinib-induced interstitial pneumonia were preliminarily analyzed. The results are presented in Table II (7–29).

## Discussion

Gefitinib is an oral selective inhibitor of the EGFR tyrosine kinase and may be effective in patients with advanced non-small-cell lung, ovarian, breast, head and neck or colon cancers. A significant survival benefit has been demonstrated for patients of Asian origin and non-smokers. At present, gefitinib is used as the second or third-line therapy for patients with locally advanced or metastatic NSCLC, following the failure of platinum and docetaxel-based chemotherapies, in a number of eastern Asian countries (30).

The most common adverse effects associated with the use of gefitinib are acneiform skin rashes, diarrhea and nausea, which are usually mild in severity and manageable (31). However, in addition to these effects, gefitinib-induced acute interstitial pneumonia is an infrequent but potentially lethal adverse effect. The precise mechanism of gefitinib-induced interstitial pneumonia remains unknown. Members of the EGF family have been demonstrated to be implicated in the repair of pulmonary damage (32). Therefore, inhibition of EGFR-mediated signaling by gefitinib may impair the repair of the bronchioloalveolar epithelium, thereby exacerbating lung injury, particularly in patients with pulmonary comorbidities (33). This may be one of the causes of gefitinib-induced ILD.

Although gefitinib-induced lung injury has a low incidence, the number of patients with gefitinib-induced lung injury is likely to increase. The incidence of ILD during gefitinib treatment varies among different ethnicities. The highest cumulative incidence of 4%, following 12 weeks of gefitinib treatment, was described in a large Japanese cohort of >1,800 patients (34). In the rest of the world, including Taiwan, the incidence of ILD has been reported to be 1% (35).

The clinical manifestations of gefitinib-induced ILD consist of chest tightness, shortness of breath, progressive dyspnea, severe hypoxemia and respiratory failure. Gefitinib-induced ILD may be diagnosed according to previously published and generally accepted clinical criteria (36). In brief, a patient may be diagnosed with gefitinib-induced ILD if the following conditions are met: (i) There is a new onset of respiratory symptoms during gefitinib treatment; (ii) there are characteristic signs on the chest radiography or CT scan, such as non-specific areas with ground-glass attenuation and extensive bilateral ground-glass attenuation or airspace consolidations with traction bronchiectasis (31); (iii) exclusion of pulmonary infection and a progression of lung cancer, including lymphangitis and carcinomatosis; and (d) exclusion of radiation pneumonitis. To relieve the symptoms of gefitinib-induced ILD, there is a requirement for timely judgment and gefitinib withdrawal to be applied and for high-dose glucocorticoid (37), oxygen inhalation and anti-infective therapies to be administered.

The patient in this case report was described as being previously healthy, with a long history of smoking and a smoking index of 800. The patient had ceased from smoking for 13 years, and was suffering from bronchioloalveolar carcinoma, which had metastasized to the lungs. Following the failure of the first-line treatments of pemetrexed plus cisplatin, EGFR and K-RAS gene results showed that the patient was suitable for the targeted therapy. One month subsequent to gefitinib treatment, the double-lung metastatic carcinoma was significantly relieved. An analysis of the domestic literature published over the past few years has shown the importance of gender, pathological type and smoking history among the factors affecting response rate and prognosis. Female patients with adenocarcinoma (particularly with alveolar cell carcinoma) and no smoking history have a good prognosis and experience an improved quality of life following gefitinib treatment. However, the present case showed that satisfactory efficacy may also be achieved in male patients with adenocarcinoma and a long-term smoking history, providing the results of the genetic testing are in accordance with the indications for gefitinib. This case report indicated that the EGFR mutation status may be the ultimate decisive factor affecting the response rate and prognosis for gefitinib treatment.

Adenocarcinoma and individual squamous cell carcinoma have been revealed to be the most common types of gefitinib-induced interstitial pneumonia. A total of 14 case reports of gefitinib-induced interstitial pneumonia that have been published in Western countries were reviewed. These cases each had individual characteristics; however, the majority concerned male smokers with adenocarcinoma. In addition, nine cases of gefitinib-induced interstitial pneumonia were analyzed from the studies published between 2004 and 2011 in China. The patients in these cases shared certain features, such as predominantly being female, with no history of smoking and with adenocarcinoma and a radiation history. These features are correlated with a low risk of ILD occurrence, a high response rate and a long survival. The results of this analysis are consistent with the results of analyses of gefitinib-induced interstitial pneumonia that have been produced in Western countries (38). The time of ILD-onset has been shown to range between 2 and 60 days following gefitinib treatment (Table II).

Following treatment, the patient in the present case recovered and continued to take gefitinib under careful medical supervision. The patient was still alive, one year subsequent to the restart of the EGFR tyrosine kinase inhibitor therapy. The outcome of this case showed that the occurrence of controllable ILD is not an absolute index for the discontinuation of gefitinib administration. The benefits to the patient and the risks associated with the use of the drug require comprehensive consideration prior to the decision regarding the continuation or discontinuation of gefitinib therapy being made.

Case 18 in Table II continued gefitinib treatment following an interruption; however, severe interstitial pneumonia occurred 13 days subsequent to the restart of the treatment, and the patient died. It was presumed that the gefitinib-induced ILD in this patient was immune-related and that, following the first dose of gefitinib, antibodies were induced in the patient. When the patient took the same drug again, subsequent to the temporary discontinuation, an antigen-antibody reaction took place, resulting in the formation of immune complexes and the onset of ILD. Similar conditions were not observed in the patient in the present case, which indicated that individual differences exist in the response to gefitinib.

In conclusion, when gefitinib is used to treat advanced NSCLC, it confers a high risk of ILD in patients with progression-free survival and a significant clinical benefit in non-smokers, females, patients with adenocarcinoma and patients with no history of thoracic radiotherapy. Gefitinib therapy is an important treatment option for patients with advanced NSCLC; however, physicians should carefully decide on the indications for the use of gefitinib and other cytotoxic agents, particularly for patients with lung comorbidities. In addition, it is necessary for careful attention to be paid to the clinical respiratory symptoms of the patients and the radiographic results, particularly during the first 1–2 months following the initiation of gefitinib treatment.

## Figures and Tables

**Table I:** Test results prior to and following treatment.

Test	Prior to treatment	Following treatment
Complete blood count		
WBC (x10^9^/l)	13.47	11.1
NEUT (%)	76.00	50.74
Biochemical examination		
AST (U/l)	131	63
ALT (U/l)	181	104
GGT (U/l)	56	49
TBIL (μmol/l)	23.7	19.2
DBIL (μmol/l)	9.8	4.4
ALB (g/l)	33	23
Arterial blood		
PO_2_ (mmHg)	51.0	65.2
PCO_2_ (mmHg)	27.0	32.1
pH	7.440	7.415
ECG	sinus tachycardia	
Blood culture		(−)
GM Test	(−)	
Sputum culture	(−)	(−)
PCT test (ng/ml)	<0.5	
Phlegm fungi smear	(−)	(−)
Sputum smear		
Gram positive coccus	++/	
GNB	+/	
Phlegm fungi training	(−)	(−)

WBC, white blood cells; NEUT, neutrophils; AST, aspartate aminotransferase; ALT, alanine aminotransferase; GGT, gamma glutamyl transpeptidase; TBIL, total bilirubin; DBIL, direct bilirubin; ALB, albumin; PO_2_, partial pressue of oxygen; PCO_2_, partial pressure of carbon dioxide; ECG, electrocardiography; GM, galactomannan; PCT, procalcitonin; GNB, gram negative bacilli.

**Table II:** Clinical characteristics of gefitinib-induced interstitial pneumonia in case reports published from 2003 to 2011.

Case no.	Age (years)	Gender	Pathology	Smoking history (years)	Onset time (days post-gefitinib administration)^[ref]^	Radiation history	Prognosis
1	75	Male	Adenocarcinoma	NA	12[7]	No	Relieved
2	60	Male	Adenocarcinoma	NA	40[8]	8 Gy	Deceased
3	55	Male	Adenocarcinoma	NA	210[9]	No	Relieved
4	59	Male	Adenocarcinoma	20	23[10]	No	Relieved
5	75	Male	Adenocarcinoma	14	2[11]	No	Deceased
6	59	Male	Adenocarcinoma	No	60[12]	No	Deceased
7	41	Female	Adenocarcinoma	No	20[13]	No	Relieved
8	74	Female	Adenocarcinoma	No	5[14]	No	Relieved
9	55	Male	Adenocarcinoma	35	42[15]	No	Relieved
10	60	Female	Adenocarcinoma	No	34[16]	No	Relieved
11	57	Male	Adenocarcinoma	NA	90[17]	No	Relieved
12	74	Female	Adenocarcinoma	No	15[18]	No	Relieved
13	77	Female	Adenocarcinoma	NA	20[19]	No	Relieved
14	28	Female	Adenocarcinoma	No	25[20]	No	Relieved
15	50	Male	Adenocarcinoma	30	38[21]	60 Gy	Relieved
16	65	Male	Adenocarcinoma	40	2[22]	No	Deceased
17	73	Male	Squamous carcinoma	Long	60[23]	No	Relieved
18	81	Male	Adenocarcinoma	Long	13[24]^a^	No	Deceased
19	66	Female	Adenocarcinoma	No	55[25]	No	Relieved
20	51	Female	Adenocarcinoma	No	56[26]	No	Relieved
21	70	Male	Adenocarcinoma	No	43[27]	44 Gy	Relieved
22	58	Female	Adenocarcinoma	No	38[28]	No	Relieved
23	79	Male	Adenocarcinoma	NA	25[29]	No	Deceased

NA, information is not provided.

a Gefitinib treatment was interrupted due to gefitinib-induced ILD and then restarted; however, severe interstitial pneumonia occurred 13 days subsequent to the restart of the treatment. Cases 1–14, data from case reports published in Western countries (references present in Pubmed); 15–23, data from case reports published in China (references not present in Pubmed).
